# *In Vitro* Differentiation of Insulin Secreting Cells from Mouse Bone Marrow Derived Stage-Specific Embryonic Antigen 1 Positive Stem Cells

**DOI:** 10.22074/cellj.2016.3842

**Published:** 2016-01-17

**Authors:** Morteza Abouzaripour, Parichehr Pasbakhsh, Nader Atlasi, Abdol Hossein Shahverdi, Reza Mahmoudi, Iraj Ragerdi Kashani

**Affiliations:** 1 Department of Anatomical Sciences, School of Medicine, Tehran University of Medical Sciences, Tehran, Iran; 2 Department of Embryology, Reproductive Biomedicine Research Center, Royan Institute for Reproductive Medicine, ACECR, Tehran, Iran; 3 Cellular and Molecular Research Center, Yasuj University of Medical Sciences, Yasuj, Iran

**Keywords:** Stage-Specific Embryonic Antigen, Insulin-Secreting Cells, Cell Differen- tiation, Diabetes Mellitus

## Abstract

**Objective:**

Bone marrow has recently been recognized as a novel source of stem cells for the treatment of wide range of diseases. A number of studies on murine bone mar- row have shown a homogenous population of rare stage-specific embryonic antigen 1 (SSEA-1) positive cells that express markers of pluripotent stem cells. This study focuses on SSEA-1 positive cells isolated from murine bone marrow in an attempt to differentiate them into insulin-secreting cells (ISCs) in order to investigate their differentiation potential for future use in cell therapy.

**Materials and Methods:**

This study is an experimental research. Mouse SSEA-1 positive cells were isolated by Magnetic-activated cell sorting (MACS) followed by characteriza- tion with flow cytometry. Induced SSEA-1 positive cells were differentiated into ISCs with specific differentiation media. In order to evaluate differentiation quality and analysis, dithizone (DTZ) staining was use, followed by reverse transcription polymerase chain reaction (RT-PCR), immunocytochemistry and insulin secretion assay. Statistical results were analyzed by one-way ANOVA.

**Results:**

The results achieved in this study reveal that mouse bone marrow contains a population of SSEA-1 positive cells that expresses pluripotent stem cells markers such as SSEA-1, octamer-binding transcription factor 4 (OCT-4) detected by immunocytochem- istry and C-X-C chemokine receptor type 4 (CXCR4) and stem cell antigen-1 (SCA-1) detected by flow cytometric analysis. SSEA-1 positive cells can differentiate into ISCs cell clusters as evidenced by their DTZ positive staining and expression of genes such as *Pdx1* (pancreatic transcription factors), *Ngn3* (endocrine progenitor marker), *Insulin1* and *Insulin2* (pancreaticβ-cell markers). Additionally, our results demonstrate expression of *Pdx1* and *Glut2* protein and insulin secretion in response to a glucose challenge in the differentiated cells.

**Conclusion:**

Our study clearly demonstrates the potential of SSEA-1 positive cells to differentiate into insulin secreting cells in defined culture conditions for clinical ap- plications.

## Introduction

Diabetes is a global health problemand one of the leading causes of morbidity and mortality in many countries, and its frequency has beensteadily increasing worldwide (1). Transplantation of islet cells can replenish those destroyed in patients with type I diabetes and can restore normoglycemic levels, but donor organs are very limited and thus waiting time is long (2). For this reason, a great deal of attention has been focused on the potential of bioengineered insulin secretingcells (ISCs) (3). Recent study have revealed the feasibility of generating ISCs obtained from embryonic stem cells; however, results are not yet satisfactory and some obstacles remain unsolved, such as immune rejection, tumor formation, source limitation, and ethical concerns about use (1).

A number of studies have suggested that bone mesenchymal stem cells (BMSCs) can differentiate into insulin secreting cells in Dulbecco’s Modified Eagle Medium (DMEM) supplemented with certain growth factors and inducer agents (1, 2). However, despite BMSCs potential to differentiate, applications using BMSCs have not been successful due to the lesser number of nucleated cells obtained from large samples and a decreased maximum lifespan after differentiation into insulin secreting cells (4). Recently, using murine bone marrow, Kuica et al. (5) have identified a homogenous population of rare stage-specific embryonic antigen 1 (SSEA- 1) positive cells. Using reverse transcription polymerase chain reaction (RQ-PCR) and immunohistochemistry these cells were shown to express marker of pluripotent stem cells such as C-X-C chemokine receptor type 4 (CXCR4), stem cell antigen-1 (SCA-1), octamer-binding transcription factor 4 (OCT-4) and NANOG.

However, experimental evidence from several studies indicates that the contribution of bone marrow derived stem cells to organ/tissue regeneration may be explained not by BMSCs but rather by the presence of SSEA-1 positive stem cells in bone marrow (5, 6). This study takes advantage of SSEA-1 positive cells isolated from murine bone marrow in an attempt to differentiate them into ISCs and investigate their differentiation potential. It will also determine whether the method might have potential as viable and effective method of cell therapy in the future.

## Materials and Methods

### Isolation of stage-specific embryonic antigen 1 positive cells from bone marrow

This study is an experimental research. All animals were treated according to guidelines, approved by Tehran University of Medical Sciences Ethics Committee under the code number 90-03-33-15668. Bone marrow aspirate was collected from the femur and tibia of 2–4 weeks old C57BL/6 mice (Pasteur Institute, Iran) by vigorous flushing with DMEM (Invitrogen, CA). Erythrocytes were eliminated using erythrocyte lysing solution (Dako, Denmark). Magneticactivated cell sorting (MACS, Abcam, USA) was used to separate the suspension of bone marrow mononuclear cells (BMMNCs) according to the expressed cell surface marker SSEA-1. BMMNCs were resuspended in cold MACS buffer (Miltenyi Biotec, Germany) and incubated with microbeadconjugated anti-SSEA-1 antibody (Miltenyi Biotec, Germany) for 45 minutes at 4˚C on a plateshaker. The cells were then washed three times with MACS buffer.

For the sorting of labeled SSEA-1 positive cells, were loaded on to a sterile LS column (Miltenyi Biotec, Germany) installed in a magnetic field (Miltenyi Biotec, Germany). The column was rinsed several times with MACS buffer and negative unlabelled cells (flow-through or negative fraction) were passed through and collected. Trapped cells (positive or labelled fraction) were washed with MACS buffer after removal of the column from the magnetic field. To achieve higher purity, the positive fraction was reloaded on a second column and the positive selection repeated, with the sample finally being collected by centrifugation. Viability and purity of the isolated SSEA-1 positive cells were evaluated by Trypan Blue (Sigma-Aldrich, USA) exclusion and flow cytometry analysis (FACSCalibur TM, Becton Dickinson, CA, USA) respectively. For flow cytometry analysis, SSEA-1 positive cells were washed with phosphate buffered saline (PBS, Sigma-Aldrich, Germany) and resuspended in PBS plus 2 % fetal calf serum (FCS, Sigma- Aldrich, Germany). The antibody staining was performed following standard protocols. 250,000 cells were washed, incubated for 45- 60 minutes with primary conjugated antibodies, washed three times, and analyzed using Becton- Dickinson flow cytometer (FACScan BD FACS Calibur, Becton-Dickinson, USA). For every sample at least 20,000 events were counted and the acquired data analysed using WinMDI 2.9 software. The following antibodies were used: anti-CXCR4 (Burlingame, CA), anti– SCA-1 (Abcam, UK) and and anti-cluster determinant 45 (CD45, BioLegend, UK). Primary antibodies were directly conjugated with fluorescein isothiocyanate (FITC, Sigma-Aldrich, Germany).

### Expansion and differentiation of stage-specific embryonic antigen 1 positive cells

Undifferentiated SSEA-1 positive cells were cultured on a feeder layer of mouse embryonic fibroblasts (MEF, Pasteur Institute, Iran) in DMEM with a low concentration of fetal bovine serum (FBS, Sigma-Aldrich, Germany). The expansion of SSEA-1 positive cellswas continued for 9 dayswith a change of medium every 3-4 days. Following expansion, cells were trypsinized and expanded SSEA-1 positive cellswere isolated from MEF cells by flow cytometry. SSEA-1 positive cellswere seeded at 1×106 cells per well to gelatin (Sigma-Aldrich, Germany) coated plates in DMEM/F-12 medium supplemented with 4 mM L-glutamine (Sigma- Aldrich, Germany), 4.5 g/l glucose (Sigma- Aldrich, Germany), 1% heat-inactivated FBS and 50 ng/ml of recombinant human Activin A (R<D Systems, USA). After 48 hours, the medium was exchanged and differentiation was carried out in DMEM/F12 medium with 4 mM L-glutamine, 4.5 g/l glucose, 5% heat-inactivated FBS in the presence of N2 supplement (Sigma- Aldrich, Germany), B27 supplement and 10 mM Nicotinamide (Sigma-Aldrich, Germany). Medium was changed every second day. Isletlike clusters appeared after 21days of culture.

### Dithizone (DTZ) staining

DTZ (Sigma-Aldrich, Germany) stock solution was prepared by dissolving 100 mg of DTZ in 5 ml of dimethyl sulfoxide (DMSO, Sigma- Aldrich, Germany). Cells induced for 21days were washed with PBS buffer twice. Next 1 mL PBS buffer and 10 μL DTZ stock solutions were added and the cells were stored a 37˚C incubator for 15 minutes. The crimson-red-stained clusters were examined with a phase-contrast microscope (CKX41, Olympus, Japan).

### RNA isolation and reverse transcription polymerase chain reaction (RT–PCR) analysis

SSEA-1 positive cells and new produced ISCs were washed and immediately transferred to micro tubes containing TRIzolj reagent (Qiagen, Germany). Tubes were vortexed to lyse the cells and kept at -20˚C until analysis. Total RNA was extracted using a Nucleospin RNAII kit (Macherey-Nagel, Germany). DNase treatment applied to clean up any possible genomic DNA contamination. cDNA synthesis was undertaken with Revert AidTMH Minus First Strand cDNA Synthesi Kit (Fermentas, Germany). A sample of 2.5 ml of cDNA, 16PCR buffer (AMS TM, Sinagen), 200 mM dNTPs, 1.5 mM MgCl2, 0.5 mM each of forward and reverse primers and 1 unit Taq DNA polymerase (Fermentas, Germany) was used to prepare reactionmixtures for PCR. PCR was performed with an optimized programme for each primer. Amplified DNA fragments were electrophoresed on 2% agarose gel. The gels were stained with ethidiumbromide (10 mg/ ml) and the amplified fragments photographed under Ultraviolet (UV) transilluminator (Uvidoc, UK). β-actin amplification was used as an internalgene control. RNA from mouse insulinoma cell line (MIN6B1) was used as positive control. Negative controls were mock RT with diethyl pyrocarbonate (DEPC, Fermentas, Germany) treated water instead of RNA or cDNA samples. In table 1, the primer pairs used for amplification of following genes are listed: *Pdx1* (pancreatic transcription factors), *Ngn3* (endocrine progenitor marker), *glucagon*, *Amylase* (exocrine pancreas marker), *Insulin1* and *Insulin2* (pancreatic β-cell markers).

**Table 1:** Primers used for reverse transcription polymerase chain reaction (RT-PCR) analysis


Genes	Primer sequences

*Pdx1*	F: 5´CCACCCCAGTTTACAAGCTC3´
	R: 5´TGTAGGCAGTACGGGTCCTC3´
*Ngn3*	F: 5´CTGCGCATAGCGGACCACAGCTTC3´
	R: 5´CTTCACAAGAAGTCTGAGAACACCAG3´
*glucagon*	F: 5´ACCAAGCCGTCATCTTCCAG3´
	R: 5´CGTATAGGGCACGTAGCAGG3´
*Amylase*	F: 5´CATTGTTGCACCTTGTCACC3´
	R: 5´TTCTGCTGCTTTCCCTCATT3´
*Insulin1*	F: 5´TAGTGACCAGCTATAATCAGAG3´
	R: 5´ACGCCAAGGTCTGAAGGTCC3´
*Insulin2*	F: 5´CCCTGCTGGCCCTGCTCTT3´
	R: 5´AGGTCTGAAGGTCACCTGCT3´
*β-actin *	F: 5`ATGAAGATCCTGACCGAGCG3`
	R: 5`TACTTGCGCTCAGGAGGAGC3`


### Immunocytochemistry

SSEA-1 positive cells that had differentiated into insulin secreting cells were cytospinned on to glass slides and allowed to dry for 15 minutes. Cells were then washed with PBS and fixed with 4% icecold paraformaldehyde for 10 minutes.The next step in the case of intracellular markers, was permeabilization by 0.4% Triton X-100 (Sigma-Aldrich, Germany). Non-specific binding sites were blocked by incubation of cells with 10% serum containing secondary antibody species. After two more washes in PBS, cells were incubated with optimal dilutions of primary antibodies overnight and with secondary antibodies for 1 hour. Briefly the SSEA-1 positive cells were stained with antibodies to SSEA-1 (1:400, anti-SSEA-1, BD Pharmingen, USA), OCT-4 (1:500, anti-OCT-4; Abcam, USA). SSEA-1 positive cells induced into ISCs were incubated with rabbit polyclonal anti-pancreatic-duodenal homeobox 1 (*Pdx1*, 1:400 dilution, Abcam, USA) and anti-Glucose Transporter2 (*Glut2*, 1:400 dilution, Abcam, USA) primary antibodies overnight at 4˚C. After five sequential three minute washes in PBS, appropriate secondary antibodies, FITCconjugated anti-goat IgG (1:500, Abcam, USA) and PE-conjugated anti-rabbit IgG (1:300, Abcam, USA) were used. After three washes with PBS, nuclei were counterstained with 4´, 6-diamidino- 2-phenylindole (DAPI, Roche, Germany). Slides were mounted with 50% (v/v) glycerol in PBS and checked under an Olympus BX 60 fluorescent microscope.

### Measurement of insulin secretion

Differentiated cells were seeded at 1×10^6^ cells per well in a 24 well culture plate, incubated overnight in culture media and grown for 3 hours in DMEM-low glucose (5.6 mM glucose). After 3 hours, the medium was removed and stored at –20˚C. Cells were then washed three times with PBS and incubated for 3 hours in DMEM-highglucose (25 mM glucose). Thereafter, the medium was stored at –20˚C. The stored media were then analyzed for insulin content using a direct mouse insulin enzyme-linked immunosorbent assay kit (ELISA, Invitrogen, CA) according to the manufacturer’s instructions. Non-induced SSEA-1 positive cells were used as a control.

### Statistical analysis

Data are expressed as mean ± SD. Comparisons between groups were carried out with one-way ANOVA. P<0.05 was considered statistically significant.

## Results

### Bone marrow as a home of pluripotent stage-specific embryonic antigen 1 positive cells

**Fig.1:**
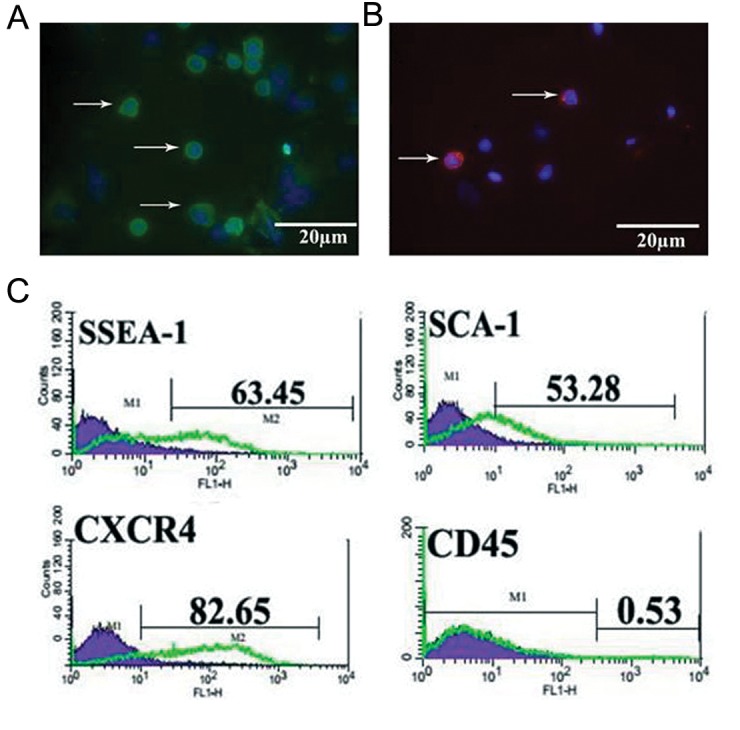
Immunostaining of pluripotency markers in bone marrow derived SSEA-1 positive cells. Expression of A. OCT-4, B. SSEA-1 in purified bone marrow derived SSEA-1 positive cells evaluated by immunofluorescence staining. Nuclei were counterstained with DAPI and C. Flow cytometric analysis of bone marrow derived SSEA-1 positive cells show that they expressed SSEA-1, CXCR4 and SCA-1 but expression of CD45 was much lower or undetectable. SSEA-1; Stage-specific embryonic antigen 1, OCT-4; Octamer-binding transcription factor 4, DAPI; 4´, 6-diamidino-2-phenylindole, CXCR4; C-X-C chemokine receptor type 4 and SCA-1; Stem cells antigen-1.

In the current study, SSEA-1 positive cells were isolated from bone marrow mononuclear cell suspension derived from 2-4 weeks old C57BL/6 mice using anti-SSEA-1 antibody and MACS. Immunofluorescence staining showed that SSEA-1 positive cells expressed embryonic stem cell markers OCT-4 (Fig .1A) and SSEA-1 at the protein level (Fig .1B).The purity of the isolated cells was determined by flow cytometery using SSEA-1, CXCR4 and SCA-1 as a surface markers of pluripotent stem cells and CD45 as a common leukocyte antigen. The flow cytometric analysis revealed that 63.45 ± 2.2% of sorted cells expressed SSEA-1, 82.65 ± 1.8% expressed CXCR4 and 53.28 ± 1.7% expressed SCA-1 but 0.53 ± 0.1% expressed CD45 (Fig .1C).

### Morphology and dithizone staining during the differentiation

**Fig.2:**
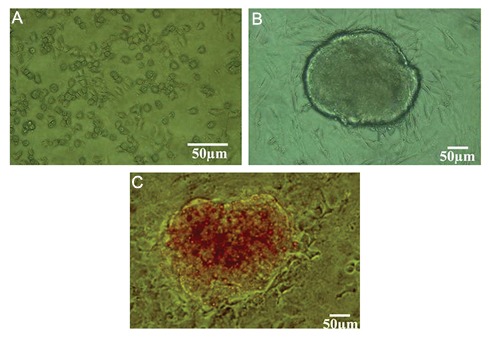
Identification of bone marrow derived SSEA-1 positive cells differentiated into insulin producing cells. A. Primary explant cultures of bone marrow derived SSEA-1 positive cells. Undifferentiated SSEA-1 positive cells were spherical in shape, B. Colony of purified SSEA-1 positive cells after culturing on MEF-coated dishes and C. Dithizone staining of insulin secreting cellsderived from bone marrow derived SSEA-1 positive cells. SSEA-1; Stage-specific embryonic antigen 1 and MEF; Mouse embryonic fibroblasts.

SSEA-1 positive cells at passages 3-5 were treated with differentiated medium and their morphologic characteristics were detected every day. Undifferentiated SSEA-1 positive cells were spherical in shape (Fig .2A). Over 14 days of induction in induction group, accumulated together and formed colony-like structures (Fig .2B). The cells aggregated in clusters and islet-like cell clusters were formed at 21 days. Derived clusters proved to be DTZ positive (Fig .2C). DTZ can specifically stain insulin granules in insulin secreting cells.

### Reverse transcription polymerase chain reaction analysis of gene expression in insulin producing cells

**Fig.3:**
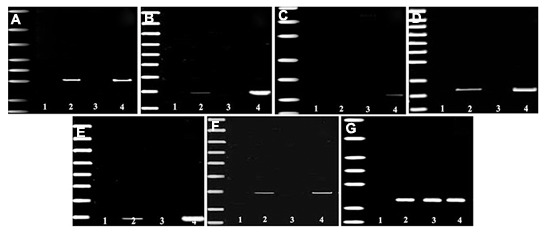
Electropherograms of RT-PCR product of mRNA extracted from bone marrow derived SSEA-1 positive cells induced into insulin secreting cells (lane 2), undifferentiated bone marrow derived SSEA-1 positive cells (lane 3) and pancreatic β-cells as a positive control (lane 4). The leftmost lane represents the DNA ladder and distilled H2O (lane 1) was used as a negative control. β-actin amplification served as an internal control. A. *Pdx1*, B. *Ngn3*, C. *glucagon*, D. *Amylase*, E. *Insulin1*, F. *Insulin2* and G. β-actin. RT-PCR; Reverse transcription polymerase chain reaction and SSEA-1; Stage-specific embryonic antigen 1.

To determine whether the SSEA-1 positive cells had differentiated into insulin producing cells, the expression of genes related to the pancreatic endocrine development and function was examined by RT-PCR analysis. As a positive control, the expression of β-actin was detected indicating our experimental system being was intact. As shown in figure 3, the results indicated that differentiated mouse SSEA-1 positive cells expressed *Pdx1*, *Ngn3*, *glucagon*, *Amylase*, *Insulin1* and *Insulin2*. However, *glucagon* and *Amylase* genes expression became silent in the cells that expressed of *Pdx1*, *Ngn3*, *Insulin1* and *Insulin2* indicating more differentiated beta-like precursor cells. These results indicate that SSEA-1 positive cells derived clusters possess the potential to differentiate into functional insulin-producing cells.

### Immunofluorescence study

**Fig.4:**
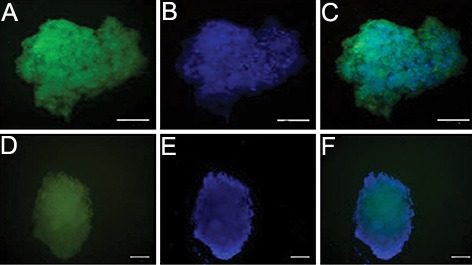
Immunofluorescence reveals bone marrow derived SSEA-1 positive cells differentiated into insulin secreting cells *in vitro*. The insulin secreting cells at days 24 were fixed and stained with antibodies against *Pdx1* and *Glut2* and visualized with secondary antibody (FITC). 4′, 6-diamidino-2-phenylindole (DAPI) was used to counter-stain DNA (blue) (scale bar; 50 μm). A. Anti*Pdx1* immunofluorescence, B. Nuclear counterstain with DAPI, C. Merged image of A and B, D. AntiGlut2 immunofluorescence, E. Nuclear counterstain with DAPI and F. Merged image of D and E. SSEA-1; Stage-specific embryonic antigen 1.

To examine the efficiency of pancreatic progenitor formation, *Pdx1* and *Glut2* expression was examined by immunofluorescence staining, represented as green dots in the immunofluorescence assay.Immunofluorescence staining showed that the *Pdx1* and *Glut2* expression was positive (Fig .4).

### Insulin content

**Fig.5:**
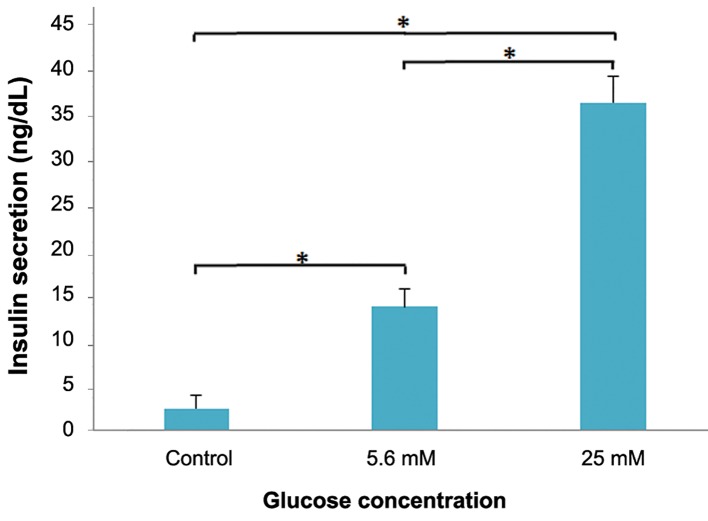
Insulin released in response to low glucose (5.6 mM) and high glucose (25 mM) stimulation in culture medium of insulinsecreting cells generated from bone marrow derived SSEA-1+ SCs (day 24) was measured. Undifferentiated bone marrow derived SSEA-1 positive cells were used as a control. Each value represents mean ± SD of three experiments. Statistical significance was tested by a Student’s t test. *; P<0.05 when compared with control and SSEA-1; Stage-specific embryonic antigen 1.

To determine whether the SSEA-1 positive cells induced into insulin secreting cells were capable of insulin secretion, we incubated them with DMEMlow glucose (5.6 mM glucose) or high (25 mM glucose) glucose and assayed culture supernatants for mouse insulin, as described earlier. As shown in figure 5, SSEA-1 positive cells induced into insulin secreting cells secreted insulin in response to glucose stimulation.

## Discussion

*In vitro* generation of ISCs, responsible for insulin synthesis, storage and release, from adult stem cells is an important advance for use in future therapies for diabetes mellitus (7). Here we demonstrate the existence of a distinct population of stem cells within murine bone marrow that expressed higher levels of the pluripotent stem cell markers, OCT-4, SSEA-1, CXCR4 and SCA-1, and have a capacity for differentiation into ISCs *in vitro*.

Previous studies demonstrate that adult tissues contain a population of stem cells that express embryonic stem cells markers, such as SSEA-1 (8, 9). The presence of these stem cells in adult tissues including bone marrow supports the hypothesis that adult bone marrow contains pluripotent stem cells deposited in embryogenesis during gastrulation (9). In this study we show that SSEA-1 positive cells could successfully differentiate into ISCs and the resultant cells were morphologically similar to pancreatic islet cells. In addition, they could also express insulin as confirmed by DTZ staining. Furthermore, using RT-PCR analyses, we observed that they expressed transcriptions factors related to pancreatic endocrine development and function, including pancreatic transcription factors Previous studies demonstrate that embryonic stem cells (ESCs) (3) and MSCs (1, 2, 4) could successfully differentiate into ISCs and the resultant cells were morphologically similar to pancreatic islet cells. In addition, they could also express insulin that was confirmed by DTZ staining. Furthermore, using RT-PCR analyses, we observed that they expressed transcriptions factors related to pancreatic endocrine development and function including *Pdx1*, *Ngn3*, *Insulin1* and *Insulin2*.

Three different protocols for differentiating ESCs into ISCs have been published during the past decade (10-12). Lumelsky et al. (10) proposed a five step protocol to differentiate mouse ESCs into ISCs. The protocol is based on the similarities between the pancreas and brain. Microarray analysis revealed that cells differentiated following this protocol failed to demonstrate increased expression of β-cell specific functional genes such as islet amyloid polypeptide, *Insulin1* and *Insulin2* (13). In the Hori protocol 10-30% of ESCs expressed Insulin, 10-20% expressed c-peptide and the remaining ESCs expressed *glucagon* which might serve as a negative marker of β-cells (11). Blysckuk et al. (12) published a protocol in which the total duration of differentiation was 48 days. All of these protocols were based on the formation of embryoid bodies’ in hanging drop culture (13).

Here, we tested a two-step differentiation protocol (14) in which mouse SSEA-1 positive cells were first cultured with activin A to favor formation of definitive endoderm in monolayer culture. Secondly this monolayer endoderm was induced to differentiate into ISCs using nicotinamide, N2 supplement and B27 supplement. This protocol is a simple modification of complex protocols for ESCs differentiation into ISCs. Using activin A to induce the differentiation of ES cells into endoderm is an important element common to almost all of the methods (15). Activin A is a dimeric glycoprotein showing high sequence homology with transforming growth factor-beta (TGF-beta) which has been shown to induce definitive endoderm cells dependent on concentration, culture conditions and time of application (14). In most recent studies, addition of activin A at the beginning of *in vitro* ESCs differentiation can cause further differentiation into definitive endodermal cells (16). Differentiation of ESCs in activin A containing medium resulted in upregulation of *Pdx1*, a marker of pancreatic progenitors, whereas medium without activin A induced ectoderm-specific *Pdx1* and Sox1 expression (14). The transcription factor *Pdx1* is critical to both β-cell development and function (17). *Pdx1* regulates the expression of a number of β-cell genes including insulin, the β-cell *Glut2* and glucokinase (18).

Nicotinamide is the precursor for the coenzyme β-nicotinamide adenine dinucleotide (NAD+). It is important for the genesis of nicotinamide adenine dinucleotide phosphate (19) and plays a significant role during the enhancement of cell survival as well as cell longevity (20). Nicotinamide, in addition to governing intrinsic cellular integrity, regulates cellular lifespan (19). Liu and Lee (16) showed that nicotinamide enhances the *in vitro* differentiation of cultured human pancreatic cells, favoring the expression of *INSULIN* and *SOMATOSTATIN*.

Previous reports have shown that the addition of N2 and B27 supplement to cultured medium, in addition to essential growth factors, play an important role in promoting sphere formation and sustaining the propagation of spheres (21). In addition, Qin et al. (22) demonstrated that N2 and B27 supplements contain many antioxidants that can help reduce the free radicals produced by oxygen and thus reduce the stress on stem cells induced by oxygen (23). Nicotinamide, activin-A, N2 and B27 supplements can promote fetal *Insulin* secreting cell differentiation and increase β-cell quantity (24).

Our results revealed that *Pdx1* was expressed in differentiated cells both at mRNA and protein levels. *Pdx1* is a transcription factor necessary for ISC maturation (25): developing ISCs co-express *Pdx1* and *Insulin*, a process that results in the silencing of V-maf musculo aponeurotic fibrosarcoma oncogene homolog B (MafB) and the expression of MafA a necessary switch in maturation of ISCs (26). *Pdx1* appears also to play a role in the fate of endocrine cells, encoding for insulin and somatostatin, two pancreatic endocrine products, while repressing *glucagon*. Thus, *Pdx1* expression apparently favors the production of ISCs rather than *glucagon* secreting cells (27). In addition to roles in ISCs differentiation, *Pdx1* is required for ISCs survival. Cells with reduced *Pdx1* have an increased rate of apoptotic programmed cell death (28).

We also detected the expression of *Glut2* in cells after 21 days of induction, using immunofluorescence. *Glut2*, a glucose transporter with the lowest affinity and the highest capacity for glucose, is expressed in pancreatic β-cell (29). *Glut2* catalyzes glucose uptake by β-cells. This is the first step in signaling a cascade, leading to glucose-stimulated insulin secretion (30). *Glut2* is present in β-cell, but not in the other islet endocrine cells (31).

These findings suggested that SSEA-1 positive cells might have the ability to secrete insulin in response to glucose. We tested this latter prediction by analyzing the secretion of insulin in response to glucose stimulation, which is an important indicator of functional pancreatic β-cells. Therefore, it was demonstrated that SSEA-1 positive cells derived ISCs are able to respond to glucose concentration and behave as matured insulin producing cells.

## Conclusion

Our study clearly demonstrates the potential of SSEA-1 positive cells to differentiate into insulin secreting cells in defined culture conditions. This finding has the potential for clinical application in the form of a new procedure for diabetes stem cell therapy. However, further research is needed to test the differentiated cells we obtained in an *in vivo* evaluation of glucose levels in a diabetic model.
